# Integrated Assessment of Pharmacological and Nutritional Cardiovascular Risk Management: Blood Pressure Control in the DIAbetes and LifEstyle Cohort Twente (DIALECT)

**DOI:** 10.3390/nu9070709

**Published:** 2017-07-06

**Authors:** Christina M. Gant, S. Heleen Binnenmars, Else van den Berg, Stephan J. L. Bakker, Gerjan Navis, Gozewijn D. Laverman

**Affiliations:** 1 Department of Internal Medicine/Nephrology, Ziekenhuisgroep Twente, Zilvermeeuw 1, 7609 PP Almelo, The Netherlands; c.gant@zgt.nl (C.M.G.); s.h.binnenmars@umcg.nl (S.H.B.); 2 Department of Internal Medicine, Division of Nephrology, University of Groningen, University Medical Center Groningen, Hanzeplein 1, 9713 GZ Groningen, The Netherlands; e.van.den.berg@umcg.nl (E.v.d.B.); s.j.l.bakker@umcg.nl (S.J.L.B.); G.j.navis@umcg.nl (G.N.)

**Keywords:** Type 2 diabetes mellitus, blood pressure, pharmacological management, nutrition, dietary sodium intake

## Abstract

Cardiovascular risk management is an integral part of treatment in Type 2 Diabetes Mellitus (T2DM), and requires pharmacological as well as nutritional management. We hypothesize that a systematic assessment of both pharmacological and nutritional management can identify targets for the improvement of treatment quality. Therefore, we analysed blood pressure (BP) management in the DIAbetes and LifEstyle Cohort Twente (DIALECT). DIALECT is an observational cohort from routine diabetes care, performed at the ZGT Hospital (Almelo and Hengelo, The Netherlands). BP was measured for 15 min with one minute intervals. Sodium and potassium intake was derived from 24-hour urinary excretion. We determined the adherence to pharmacological and non-pharmacological guidelines in patients with BP on target (BP-OT) and BP not on target (BP-NOT). In total, 450 patients were included from August 2009 until January 2016. The mean age was 63 ± 9 years, and the majority was male (58%). In total, 53% had BP-OT. In those with BP-NOT, pharmacological management was suboptimal (zero to two antihypertensive drugs) in 62% of patients, and nutritional guideline adherence was suboptimal in 100% of patients (only 8% had a sodium intake on target, 66% had a potassium intake on target, 3% had a sodium-to-potassium ratio on target, and body mass index was <30 kg/m^2^ in 35%). These data show pharmacological undertreatment and a low adherence to nutritional guidelines. Uncontrolled BP is common in T2DM, and our data show a window of opportunity for improving BP control, especially in nutritional management. To improve treatment quality, we advocate to incorporate the integrated monitoring of nutritional management in quality improvement cycles in routine care.

## 1. Introduction

Type 2 Diabetes Mellitus (T2DM), with an estimated number of 422 million patients worldwide, is one of the major conditions associated with cardiovascular events and cardiovascular death [1]. Therefore, the prevention of the development and progression of such complications is a main goal in the treatment of T2DM, and evidence-based recommendations to reach this goal are incorporated in treatment guidelines. Treatment consists of pharmacological and non-pharmacological management, the latter consisting in large part of nutritional guidance. Still, cardiovascular complications develop in the majority of T2DM patients, demonstrating the large challenge of adequate treatment [2,3]. One explanation for this could be a failure to reach guideline treatment targets. Indeed, several studies have shown that targets for blood pressure, glycemic control, and Low Density Lipoprotein (LDL) cholesterol are not reached in a large number of patients [4,5,6,7,8].

Pharmacological and nutritional management are often studied as separate entities, despite the fact that both are crucial elements of treatment. We hypothesize that a systematic assessment of both pharmacological and nutritional management can identify targets for the improvement of treatment quality. The DIAbetes and LifEstyle Cohort Twente (DIALECT) cohort study was specifically designed for this purpose. DIALECT is an observational study in T2DM patients in a well-defined region in The Netherlands, and uses validated and detailed real-world data on nutritional habits, pharmacological treatment, and current clinical condition. To obtain non-biased data on individual nutrient intake, 24-hour urine collections were used and stored in a biobank to allow for future analyses [9].

We aim to address how well the targets for blood pressure management are reached, and how this is related to (1) pharmacological management; and (2) nutritional management (i.e., the dietary intake of salt [10,11], potassium [12,13], body mass index (BMI), and alcohol). Moreover, we assessed additional nutritional parameters for which no specific counselling was given, but have been shown to be relevant to cardiovascular risk in diabetic kidney disease (magnesium [14,15,16] and phosphate [17,18]). Because the presence of diabetic kidney disease implicates different blood pressure targets, we analysed patients without and with renal involvement separately.

## 2. Materials and Methods

### 2.1. Study Design and Participants

DIALECT is a prospective cohort study in patients with T2DM, performed in the ZGT Hospital, which is located in Almelo and Hengelo, The Netherlands. It is designed to study pharmacological and non-pharmacological management in a regional T2DM population treated in a secondary health care center. All patients with T2DM and aged 18+ years treated in the outpatient clinic of our hospital were eligible, with the only exclusion criteria being an inability to understand the informed consent procedure, insufficient knowledge of the Dutch language, or a dependency on renal replacement therapy.

This paper reports on the DIALECT-1 population, consisting of the first 450 patients, recruited between September 2009 and January 2016. The inclusion of new patients in DIALECT-2 will be performed until December 2019, or until the number of 850 is reached. The study is performed according to the guidelines of good clinical practice and the Declaration of Helsinki. It has been approved by the local institutional review boards (METC-registration numbers NL57219.044.16 and 1009.68020), and is registered in the Netherlands Trial Register (NTR trial code 5855).

### 2.2. Study Procedures

**Figure 1:**
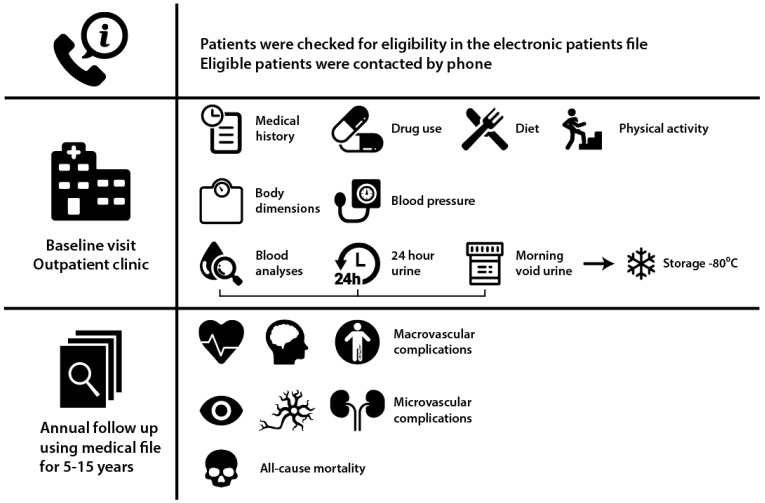
Patient inclusion and data collection.

Patients were screened for eligibility in the electronic patient file, and subsequently invited for a study visit. At the clinic, all of the information relevant to the medical condition was recorded in a database (Figure 1, Supplementary Table S1). Height, weight, and waist and hip circumference were measured. Body mass index was calculated as weight divided by height squared (kg/m^2^), and body surface area was estimated by applying the universally adopted formula of DuBois [19]. Blood pressure was measured in a supine position by an automated device (Dinamap^®^; GE Medical systems, Milwaukee, WI, USA) for 15 min with a one-minute interval. The mean systolic and diastolic pressure of the last three measurements was used for further analysis.

Physical activity was assessed using the Short Questionnaire to Asses Health enhancing physical activity (SQUASH) questionnaire, which was previously validated in [20]. The 24-hour urinary content of specific substances was measured where possible and appropriate.

Routine laboratory tests were performed in venous blood, including blood count tests, liver function tests, renal function tests, HbA1c, and cholesterol. The estimated glomerular filtration rate (eGFR) was calculated using the Chronic Kidney Disease Epidemiology Collaboration (CKD-EPI) formula [21]. From samples of a 24-hour urine collection, the following parameters were measured: sodium, potassium, creatinine, calcium, phosphate, chloride, albumin, protein, urea, and uric acid excretion. Twenty-four-hour urinary excretion was calculated by multiplying these concentrations with the volume of the 24-hour urine collection. Creatinine clearance was calculated from the 24-hour urine creatinine excretion and the plasma creatinine concentration. For the proper collection of the 24-hour urine sample, patients were instructed to dispose of the first morning void urine, and thereafter collect all urine in the provided canister until the first morning void urine of the next day. In between voids, they were instructed to store the canister in a dark cool place, preferably in a refrigerator. A separate single morning void urine was used to assess the urinary albumin-to-creatinine ratio.

The samples of blood, 24-hour urine collection, and morning void urine were stored in a biobank at −80 degrees Celsius for additional analyses, as specified in Supplementary Table S1.

### 2.3. Routine Clinical Care

Diabetes care in the Netherlands is standardised, both in the outpatient clinic and at the general practitioner. It consists of three to four outpatient clinic visits per year. The development of albuminuria is assessed yearly using the albumin–creatinine ratio in a single morning void urine. Retinopathy is assessed at one to two year intervals. Neuropathy is assessed yearly using monofilament and vibration tests with a tuning fork.

Lifestyle management in T2DM consists of guidance regarding weight loss, increasing physical activity, and smoking cessation, and of referral to a dietician for dietary guidance on weight loss and the adherence to dietary guidelines, including sodium restriction and stimulating an intake of fruit and vegetables. The frequency of dietary follow-up visits is targeted at the individual goals and needs of patients depending on personal preferences as well as comorbidity. At each doctor visit, target HbA1c and blood pressure are monitored, and pharmacological intervention is adjusted accordingly. Cholesterol levels are monitored yearly. Targets for HbA1c and LDL cholesterol are often individualized; the general targets are <53 mmol/L and <2.5 mmol/L, respectively.

### 2.4. Definitions

The blood pressure (BP) targets in our analyses were derived from the international guidelines for diabetes management, which have been adopted for use in The Netherlands [22,23]. In patients with diabetic kidney disease, the BP target was set according to the Kidney Diseases Improving Global Outcomes (KDIGO) guidelines, which are internationally acclaimed guidelines for chronic kidney disease, and are also applied in The Netherlands [23]. Patients with diabetic kidney disease without albuminuria (eGFR <60, no albuminuria) had a BP target of ≤140/90 mmHg, while patients with albuminuria and either an eGFR ≥60 mL/min or an eGFR <60 mL/min had a BP target of ≤130/80 mmHg. For patients with T2DM without diabetic kidney disease, the European Association for the Study of Diabetes (EASD) guidelines are used, which stipulate a blood pressure (BP) target of <140/85 mmHg [22]. Accordingly, the patients were grouped by eGFR above or below 60 mL/min and by the presence of albuminuria. Albuminuria was defined as a 24-hour urinary albumin excretion >30 mg/day. As the EASD and KDIGO guidelines for those without albuminuria differ slightly, we performed all of the analyses using the EASD guidelines for those with eGFR <60 and no albuminuria as well. The results were virtually similar, and for the sake of conciseness, the data is not shown.

The targets for nutritional management were set according to the Dutch guidelines when available. The target dietary salt intake was ≤6 g/day [24], and the target dietary potassium intake was set at ≥3.5 g/day, according to best evidence [13]. The target alcohol intake was ≤2 units per day for women, and ≤3 units per day for men. It should be noted that in 2015, the Health Council of The Netherlands changed the guidelines for alcohol consumption to zero units per day; however, our patients were included in the study before the introduction of these new guidelines [25]. The target BMI was <30 kg/m^2^. The target for smoking was either no smoking history, or having previously stopped smoking.

The data on dietary intake of salt, potassium, and proteins were derived from 24-hour urinary excretion. For this, it is important to realise that the patients in our cohort were assessed under steady state conditions, in which the net renal excretion of sodium is almost equal to the dietary intake of sodium, with only approximately 5–10% being excreted by other routes (e.g., sweat or feces) [9]. Therefore, 24-hour urinary sodium excretion is considered the gold standard for the assessment of sodium intake [9,26], and dietary salt intake was calculated by multiplying the net 24-hour sodium excretion (in mol/day) with the molar weight of salt (NaCl, 58.44 g/mol). Dietary potassium intake was calculated from urinary potassium excretion under the assumption of a renal excretion rate of 77% [13,27]. Dietary protein intake was calculated from urinary urea nitrogen excretion using the Maroni formula [28]. As the renal excretion of magnesium is lower in patients with a low eGFR, dietary magnesium intake could not be calculated from urinary magnesium excretion with the same formula (using the assumption of an intestinal absorption of 30%) [16]. Therefore, we present the urinary daily excretion of magnesium. Also, while no consensus exists to calculate dietary phosphate intake from the urinary excretion, urinary phosphate excretion does reflect variability in intestinal phosphate uptake [29,30], so we present the urinary excretion values.

### 2.5. Statistical Analyses

All of the statistical analyses were performed using Statistical Package for the Social Sciences (SPSS), version 23.0. Normally distributed data are presented as mean ± standard deviation. Skewed variables are expressed as median [interquartile range]. Dichotomous variables are presented in number and percentage. First, we divided the population according to the presence of albuminuria and/or a reduced eGFR (<60 mL/min), as in these groups the target BP is different (<140/85 for those without diabetic kidney disease, ≤140/90 mmHg for patients without albuminuria and an eGFR <60 mL/min, and ≤130/80 mmHg for those with albuminuria). Second, we divided the population into two groups, according to the reached blood pressure. These groups are denoted as “Blood pressure on target” (BP-OT) and “Blood pressure not on target” (BP-NOT), respectively. The differences between the groups were analysed using the student *t-*test, one-way ANOVA, the Mann–Whitney U test, the Kruskall–Wallis test, and the Chi Square test when appropriate. To perform a multivariate analysis of the determinants of not on target BP, multivariate logistic regression was used. In order to adjust for age and gender, the differences in nutritional data among the groups were also determined using mixed model analyses with Sidak post-hoc tests.

## 3. Results

**Figure 2:**
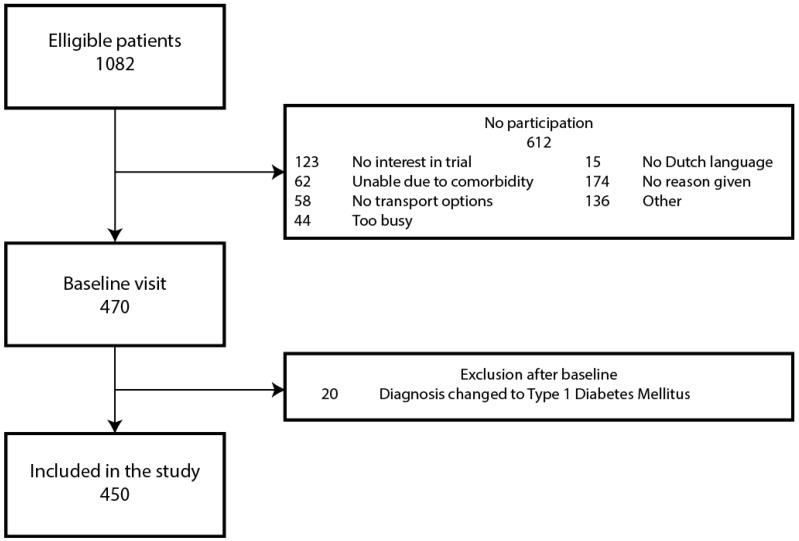
Patient recruitment flowchart.

Between September 2009 and January 2016, 1082 eligible patients were identified and invited to participate in the study, of whom 470 were enrolled in the study and performed the baseline visit. The most common causes for not participating in the study were: No interest in research, and inability due to co-morbidity (Figure 2). Twenty patients were excluded after the baseline visit, as in closer analysis their correct diagnosis was Type 1 Diabetes Mellitus instead of Type 2. All of the remaining 450 patients were included in our data analysis.

### 3.1. Baseline Pharmacological and Nutritional Characteristics

The baseline data are presented in Table 1, by a break-up according to reduced eGFR (<60 mL/min) and the presence of albuminuria. The mean age of the participants was 63 ± 9 years, and was higher in the groups with eGFR <60 (Table 1). There were more men (58%) than women, and men were over-represented in the albuminuria groups (74% and 77% respectively for eGFR ≥60 and <60). The mean BMI was 32.9 ± 6.2 kg/m^2^, reflecting a predominantly obese T2DM population, and BMI did not differ among the groups (Table 1).

There was no renal involvement in 57% of the patients (eGFR ≥60/Alb−; Table 1). Of all of the patients, 30% (*n* = 136) had albuminuria, either with a preserved (*n* = 85, eGFR ≥60/Alb+) or reduced renal function (*n* = 51, eGFR <60/Alb+). Fifty-two patients (12%) had a reduced renal function without albuminuria (eGFR <60/Alb−). The mean systolic blood pressure was 139 ± 16 mmHg, and the mean diastolic BP was 76 ± 9 mmHg. Most of the patients (81%) used one or more antihypertensive drugs. The target BP was reached in 53% of all patients, while 47% had BP not on target. In patients with albuminuria, 33% and 24% reached the target blood pressure in eGFR ≥60 and in eGFR <60, respectively (Table 1). Additionally, a blood pressure of ≤140/90 mmHg was reached in 48% and 41% of albuminuria patients with an eGFR ≥60 and eGFR <60, respectively. The group with albuminuria and eGFR <60 received the largest number of antihypertensive drugs (3 (2–4) drugs, Table 1). Additionally, the number of patients with hypertension requiring 4+ drugs was highest in this group (59%, *p* < 0.001). In contrast, the antihypertensive drug use in the eGFR ≥60/Alb+ group is not higher than in the other groups (2 (1–3) drugs). Patients without chronic kidney disease (CKD) (Table 1, group eGFR ≥60/Alb−) most commonly used renin-angiotensin-aldosterone-system inhibition (RAASi) (59%), followed by β-blockers (39%), and thiazide diuretics (32%). This was different in those with CKD (groups eGFR ≥60/Alb+, eGFR <60/Alb−, and eGFR <60/Alb+): RAASi (77%), β-blockers (62%), and Calcium antagonists (31%). There were two patients with an eGFR <60 that used a phosphate binder, one in the Alb− group, and one in the Alb+ group.

The mean dietary salt intake was high, namely, 10.9 g of salt per day, and was considerably higher in the groups with preserved eGFR. When adjusting for age and gender, these differences remained virtually similar (data not shown). In the overall population, only 53 patients (12%) adhered to the dietary guidelines for dietary salt intake, ≤6 g/day, and in the eGFR ≥60/Alb+ group this percentage was even lower, i.e., 6%. In total, 8% of patients had a salt intake of ≤5 g/day as recommended by the WHO. The mean potassium intake was 3.9 ± 1.3 g/day, and 66% of patients had an intake, as recommended, above 3.5 g/day (Table 1). The mean urinary magnesium excretion was 4.0 ± 2.1 mmol/day, and as expected was lower in patients with an eGFR <60 mL/min than in those with an eGFR ≥ 60mL/min. The mean urinary phosphate excretion was 27.5 ± 11.6 mmol/day, and the mean calculated dietary protein intake was 92 ± 27 g/day.

### 3.2. Pharmacological and Nutritional Management in BP-On Target (BP-OT) and BP-Not On Target (BP-NOT) Groups

Table 2 shows the patients’ characteristics by a break-up of BT-OT and BP-NOT. Patients with BP-NOT were more often men (64% vs. 53%, *P* = 0.018), and had a higher HbA1C (59 ± 12 vs. 56 ± 11 mmol/L, *P* = 0.031). While the presence of albuminuria was a strong predictor of uncontrolled BP (46% vs. 17%, *p* < 0.001), poor BP control was not associated with an eGFR <60 mL/min (24% vs. 22%).

Patients with BP-OT used loop diuretics more often than those with BP-NOT (Table 2). There were no other differences in the pharmacological treatment between the BP groups; neither in the types of prescribed drugs, nor in the total number of prescribed antihypertensive drugs. Surprisingly, of the patients with BP-NOT, 21% did not use any antihypertensive drug, while 20% used only one, and 21% used two antihypertensive drugs.

**Figure 3:**
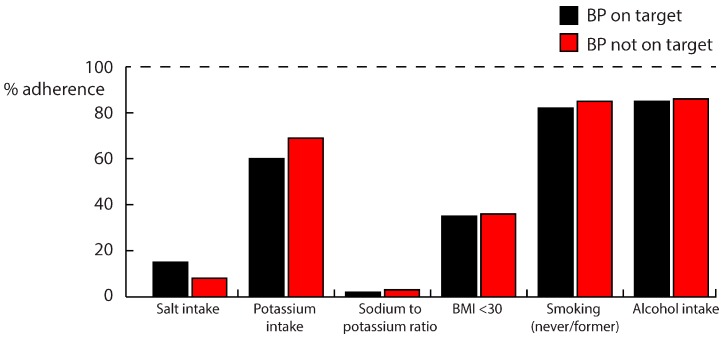
% Adherence to nutritional guidelines, by a breakup of blood pressure on target/not on target. Alcohol intake is self-reported, and the target intake was ≤2 units per day for women and ≤3 units per day for men.

Adherence to the recommended nutritional guidelines by a breakup of BP-OT and BP-NOT is shown in Figure 3. In both groups, adherence to the recommended lifestyle guidelines was poor, and the total number of lifestyle targets adhered to did not differ between the groups (3 (2–3) in BP-OT vs. 3 (2–3) in BP-NOT, *p* = 0.22). In patients with BP-NOT, 8% had a dietary salt intake below the recommended 6 g/day, which was lower than those with BP-OT (15%, *p* = 0.025). Adherence to the potassium guideline (66% of patients) did not differ among the groups. Only 3% of patients had a sodium-to-potassium ratio ≤1.0 in both BP groups. There were only three patients (1%) who adhered to both the recommended intakes of salt and potassium. BMI was ≤30 kg/m^2^ in 35% of patients, and this proportion did not differ among the BP groups. The smoking and alcohol guidelines were adhered to by 83% and 86% of all patients, and these proportions were not different among the BP groups. We found no differences in the other nutritional factors between the BP-OT and BP-NOT groups (Table 2). In the total population, there was only one patient (with BP-OT) who adhered to all of the lifestyle guidelines simultaneously. There were no differences in lifestyle guidelines adherence between those with zero to two antihypertensives and those with three-plus antihypertensives.

In the multivariate logistic regression analysis, albuminuria and the use of loop diuretics remained the only significant predictors of BP-NOT (data not shown).

## 4. Discussion

In this paper, we present the blood pressure management of Type 2 diabetes patients (T2DM), using combined data on pharmacological and nutritional management in a real-life secondary health care setting. As anticipated, the prevalence of hypertension was high, and 81% of patients were on antihypertensives. However, BP control was poor, as the target BP was not reached in 47% of patients. An integrated assessment of pharmacological and nutritional management demonstrated a large window of opportunity for improving BP control in T2DM, both by intensifying antihypertensive drug treatment, and increasing nutritional guideline adherence.

The proportion of patients with their blood pressure on target (BP-OT) in our cohort is in line with findings in other T2DM cohorts [31], as well as diabetic kidney disease cohorts [5,32]. The proportion was lower than found in T2DM patients treated in the primary care setting, in whom adequate blood pressure control was found in 85% of patients [33,34], which may well reflect the referral policy, with more difficult patients being referred to secondary care. In the baseline data of the LEADER-4 trial (a randomized clinical trial in T2DM patients) [31], where 51% of patients had BP-NOT, the antihypertensive use was lower than we report, as about 80% of all patients used zero to two antihypertensive drugs. In diabetic kidney disease studies (i.e., studies with T2DM and either albuminuria and/or eGFR <60), the antihypertensive drug use is mostly in line with our findings, with >90% of patients using antihypertensive drugs, and RAASi being the most frequently used drug [5,32]. In line with our findings, Smits et al. found that RAASi was the most commonly used class of antihypertensive drugs in a Dutch T2DM cohort in primary care, followed by betablockers and diuretics [33]. The number of used antihypertensive drugs they report is largely comparable to our findings, albeit that the use of four to five drugs seems more common in our secondary care cohort. It should be noted that in these studies regarding BP control in a real world setting, data regarding nutrient intake is not available.

An important issue when evaluating the pharmacological management of blood pressure is treatment non-adherence, which reportedly ranges from 31 to 40% in patients with poorly controlled blood pressure [35,36]. Thus, establishing an infrastructure that allows the monitoring of adherence would be of great value. Yet, even assuming a drug-treatment non-adherence rate of 40% in our patients, the non-adherence to lifestyle measures seems to stand out as an additional important target for intervention.

What can be done to improve BP control in T2DM? Our data, and data from other trials, clearly show that true therapy-resistant hypertension, defined as hypertension persisting despite three antihypertensive drugs at maximum tolerable dosage of which one is a diuretic, is not the issue in most patients with BP-NOT. The majority of BP-NOT patients (62%) do not use more than two antihypertensive drugs, illustrating the opportunity for intensifying pharmacological treatment. One promising option in this regard is the removal of excess extracellular fluid with diuretics, especially in those patients with a high salt intake.

Our data show that, especially for nutritional management, there is a large window of opportunity for improvement, as in the total population only one patient adhered to all of the nutritional guidelines simultaneously. This is highly relevant, since lifestyle interventions have the potential to not only reduce BP, but to also reduce the overall cardiovascular risk [11,37,38,39,40]. Even though dietary counselling has already been part of their routine care, the mean daily salt intake in our population was almost 11 g/day, roughly twice that of the recommended 6 g/day, and considerably higher than the mean salt intake in the general Dutch population of 8.5 g/day [41]. Previously, Mente et al demonstrated that for each 1-gram increment in estimated sodium excretion, blood pressure was 2/1 mmHg higher, where this slope was more pronounced and steeper in those with hypertension, high-sodium diets (>5g/day), and older persons [42]. Therefore, the most obvious step to improve non-pharmacological management would be to reduce dietary salt intake. This is underscored by a previous study, performed in T2DM patients in the same region, which has shown that, although the aim to reduce dietary salt intake to <6 g/day was not reached, even a relatively modest reduction in salt intake from 12 to 9 g/day can reduce blood pressure by 6/3 mmHg and albuminuria by 42% while under RAASi [43]. Furthermore, reducing salt intake is associated with potentiating the antihypertensive effects of RAASi [44,45,46].

There is evidence that a combined dietary approach aimed at reducing salt while increasing potassium intake has the potential to improve cardiovascular risk management [12,47]. However, the potassium intake in our patients was generally already above the recommended intake of 3.5 g/day. Therefore, the finding that the sodium-to-potassium ratio was higher than the deemed optimal ratio of 1 mmol/mmol in 97% of patients is primarily determined by high salt intake.

To improve blood pressure control, dietary intervention could also be aimed at reducing body weight. The mean BMI in our cohort was above 30 kg/m^2^. While a relationship between obesity and blood pressure has previously been demonstrated [7,48], we did not find such an association here. This might be due to the fact that we had few participants with a BMI < 25 kg/m^2^, and therefore did not have a large enough dispersion to differentiate between the BP groups. Intentional weight loss has been associated with beneficial effects, both on BP and on other cardiovascular risk factors such as LDL cholesterol and glycemic control [49]. Therefore, even though weight loss is notoriously difficult to achieve, especially in patients on insulin treatment, it should remain a priority in the non-pharmacological treatment of T2DM, and also in secondary health care centres.

Finally, an association between the intake of magnesium and phosphate and blood pressure has been reported previously [14,17,18,50,51]. Here, we did not find differences in urinary magnesium excretion or in urinary phosphate excretion between those with BP-OT and BP-NOT. As the urinary excretion of magnesium and phosphate is lower in those with a low eGFR, these results might be misleading. However, the proportion of patients with a low eGFR was similar in both the BP-OT and BP-NOT groups, making it less likely that differences in the urinary excretion of magnesium and phosphate between the BP groups were masked by differences in urinary excretion due to a low eGFR. In the general population, a continuous relationship between lower magnesium excretion and the risk for hypertension was reported [16]; moreover, patients with a low magnesium intake had a greater risk of developing ischemic heart disease [52]. While no nutritional recommendations are currently available for magnesium to stratify adequate/inadequate intake, in our population approximately 28% of patients had a magnesium excretion below the values associated with ischemic heart disease in the general population. Regarding phosphate intake, population-based studies as well as studies in CKD have shown associations with outcome, albeit not equivocal [53,54], and it has been proposed that excess phosphate intake is a risk factor that is generally overlooked in patients with early stages of CKD by lack of measurements [55]. While more research is needed on the relation between magnesium and phosphate excretion and adverse outcomes, our data illustrate that 24-hour urine, collected to assess the intake of established nutritional targets such as salt and potassium, can simply be used to establish a more complete nutritional profile, which could be useful for future improvements in nutritional studies and counselling.

It should be noted that the adherence to nutritional guidelines was equally poor in the BP-OT and BP-NOT groups. While in the BP-NOT group there is more urgency to adhere to these guidelines, namely to correct BP, the adherence to the guidelines in the BP-OT group should not be overlooked. In regard to salt intake, previously it has been shown that a higher salt intake while under RAASi is associated with worse cardiovascular outcomes, even independent of BP [46]. Furthermore, as stated above, intentional weight loss has many benefits that surpass BP management [49], and therefore can also greatly improve outcomes if BP is already on target. Lastly, in a population-based cohort, low potassium intake has been associated with the occurrence of chronic kidney disease [56].

The DIALECT study has several strengths, including the use of real-world data from a cohort representative of secondary health care in T2DM, at least in the context of the Dutch referral health care setting. Second, we study the integrated role of non-biased data on both pharmacological and non-pharmacological parameters on BP, which is an important approach, as in cardiovascular risk management pharmacological and non-pharmacological interventions go hand in hand. Third, through the use of 24-hour urine collections, we provide objective measurements of dietary intake and several relevant nutrients.

There are also some limitations. An observational study cannot prove causal relationships. Also, there is some risk of response bias, although patient characteristics were similar between those who did and did not participate.

What are the implications of our study? Adequate management equals the sum of measures taken in combination with compliance. Our data on poor nutritional management do not distinguish between a lack of adequate nutritional counselling and a lack of compliance. However, it is well established that sustained lifestyle change is difficult to achieve, demonstrating that currently no modus of adequate counselling and therefore adequate management exists. The question, therefore, is how to establish this. Previous well-designed studies, using interventions of intensive nurse practitioner support and self-management, both did not lead to neither long-standing changes in nutritional habits, nor a reduction of cardiovascular outcomes [57,58]. As alternative approach, improvement strategies as tested for pharmacological management could be considered. In particular, it has been shown that the systematic evaluation of prescription quality as assessed by prescription quality indicators not only improved pharmacological compliance with guidelines, but also patient outcomes [59]. To the best of our knowledge, such approaches have never been developed and tested for nutritional management. As several objective parameters are available, such as the urinary excretion of sodium and potassium, this would be feasible in routine clinical care. Therefore, to improve blood pressure control, in our opinion, the use of nutritional quality indicators may have the potential to improve treatment quality as a whole.

## 5. Conclusions

Uncontrolled BP is common in T2DM, especially in those with microalbuminuria. An integrated assessment of pharmacological and nutritional management demonstrated a window of opportunity for improving BP treatment, especially in nutritional management. We advocate that incorporating the integrated monitoring of pharmacological and nutritional management in quality control cycles has the potential to improve treatment quality in T2DM.

## Figures and Tables

**Table 1:** DIALECT-1 Baseline, nutritional, and pharmacological characteristics.

			eGFR ≥60		eGFR <60		
Variable		Total Population	Albuminuria No	Albuminuria Yes	Albuminuria No	Albuminuria Yes	*p*-Value
Number of patients (% of population)		450	257 (57)	85 (19)	52 (12)	51 (11)	
***Patient characteristics***							
Age (years)		63 ± 9	61 ± 9	62 ± 8	67 ± 8 *^,†^	69 ± 7 *^,†^	<0.001
Male, *n* (%)		259 (58)	139 (54)	63 (74)	19 (37)	39 (77)	<0.001
Years T2DM (years)		11 (7–18)	11 (7–18)	14 (8–19)	12 (6–17)	10 (6–15)	0.45
Serum HbA1C (mmol/mol)		57 ± 12	58 ± 11	59 ± 13	54 ± 11	57 ± 13	0.15
Insulin use, *n* (%)		284 (63)	160 (62)	64 (75)	31 (60)	28 (55)	0.07
Systolic blood pressure (mmHg)		139 ± 16	136 ± 15	140 ± 19	131 ± 13 ^†^	139 ± 17	0.009
Diastolic blood pressure (mmHg)		76 ± 9	75 ± 9	76 ± 10	70 ± 9 *^,†^	75 ± 10 ^‡^	0.004
BP on target, *n* (%)		236 (53)	155 (60)	28 (33)	41 (79)	12 (24)	<0.001
Macrovascular disease, *n* (%)		158 (35)	68 (27)	36 (42)	25 (48)	31 (61)	<0.001
eGFR (mL/min)		84 (62–97)	92 (78–100)	88 (74–99)	47 (36–54)	39 (33–45)	<0.001
Albumin excretion (mg/day)		11 (3–66)	5 (2–11)	94 (62–202)	4 (1–12)	332 (93–661)	<0.001
***Pharmacological management***							
RAASi, *n* (%)		296 (67)	152 (59)	63 (74)	39 (75)	42 (82)	0.001
β-blockers, *n* (%)		207 (46)	100 (39)	37 (44)	36 (69)	33 (65)	<0.001
Thiazide diuretics, *n* (%)		137 (31)	81 (32)	15 (18)	21 (40)	18 (35)	0.02
Calcium antagonists, *n* (%)		101 (23)	43 (17)	26 (31)	13 (25)	19 (37)	0.002
Loop diuretics, *n* (%)		81 (18)	26 (10)	18 (21)	17 (33)	20 (39)	<0.001
Potassium sparing diuretics, *n* (%)		43 (10)	11 (4)	8 (9)	12 (23)	12 (24)	<0.001
Number of antihypertensives		2 (1–3)	2 (0–3)	2 (1–3)	3 (2–3)	3 (2–4)	<0.001
	No antihypertensive therapy, *n* (%)	83 (19)	65 (25)	12 (14)	1 (2)	2 (4)	<0.001
	1 drug, *n* (%)	101 (23)	61 (24)	17 (20)	6 (12)	6 (12)	
	2 drugs, *n* (%)	106 (24)	57 (22)	28 (33)	13 (25)	11 (22)	
	3 drugs, *n* (%)	91 (20)	44 (17)	15 (18)	21 (40)	12 (24)	
	4 drugs, *n* (%)	56 (13)	24 (9)	10 (12)	8 (15)	13 (26)	
	5+ drugs, *n* (%)	11 (3)	6 (2)	3 (4)	3 (6)	7 (14)	
Hypertension requiring 4+ drugs, *n* (%)		117 (26)	48 (19)	23 (27)	16 (31)	30 (59)	<0.001
Total number of drugs		7 ± 3	6 ± 3	7 ± 2	8 ± 3 *	9 ± 3 *^,†^	<0.001
***Non-pharmacological management***							
BMI (kg/m^2^)		32.9 ± 6.2	32.9 ± 6.5	32.9 ± 5.4	33.3 ± 6.2	32.3 ± 6.1	0.89
Current smoker, *n* (%)		74 (17)	41 (16)	15 (18)	10 (19)	8 (16)	0.93
Alcohol intake (units per month)		5 (0–30)	5 (0–28)	10 (0–47)	3 (0–24)	12 (0–40)	0.22
25(OH) Vitamin D (nmol/L)		42 ± 20	43 ± 18	37 ± 19	42 ± 26	44 ± 22	0.09
*Urinary excretion*							
Urinary creatinine excretion (mmol/day)		13.8 ± 4.8	13.9 ± 4.9	14.8 ± 5.4	12.8 ± 4.2	12.8 ± 3.6	0.03
Urinary magnesium excretion (mmol/day)		4.0 ± 2.1	4.1 ± 2.1	4.4 ± 2.3	3.3 ± 1.7 *^,†^	3.2 ± 1.4 *^,†^	0.001
Urinary phosphate excretion (mmol/day)		27.5 ± 11.6	28.2 ± 12.2	30.3 ± 12.6	22.7 ± 7.7 *^,†^	25.0 ± 7.9	0.001
Sodium-to-potassium ratio (mmol/mmol)		2.5 ± 1.0	2.5 ± 1.0	2.8 ± 1.2	2.2 ± 0.7 ^†^	2.3 ± 0.8	0.004
*Calculated intake*							
Dietary salt intake (g/day)		10.9 ± 4.7	11.0 ± 4.3	12.7 ± 5.6 *	8.7 ± 4.0 *^,†^	9.7 ± 3.9 ^†^	<0.001
	Salt intake ≤6 g/day	53 (12)	26 (10)	5 (6)	15 (29)	7 (14)	<0.001
Dietary potassium intake (g/day)		3.9 ± 1.3	4.0 ± 1.4	4.1 ± 1.1	3.5 ± 1.3	3.6 ± 0.9	0.01
	Potassium intake ≥3.5 g/day	290 (66)	173 (69)	62 (73)	27 (53)	29 (59)	0.06
Dietary protein intake (g/day)		92 ± 27	94 ± 28	98 ± 29	80 ± 23 *^,†^	84 ± 21 ^†^	0.001

* *p* < 0.05 vs. eGFR ≥60/Albuminuria (Alb)−; ^†^ *p* < 0.05 vs. eGFR ≥60/Alb+. ^‡^ *p* < 0.05 vs. eGFR <60/Alb−. Abbreviations: T2DM, Type 2 Diabetes Mellitus; BP, blood pressure.

**Table 2:** DIALECT-1 pharmacological and nutritional management by a breakup of BP on target/not on target.

Variable		BP On Target	BP Not On Target	*p*-Value
*Patient characteristics*		*n* = 239	*n* = 210	
Age (years)		63 ± 9	63 ± 9	0.36
Male, *n* (%)		126 (53)	134 (64)	0.02
Years T2DM (years)		11 (7–17)	12 (7–18)	0.26
Serum HbA1C (mmol/mol)		56 ± 11	59 ± 12	0.03
Insulin use, *n* (%)		149 (62)	136 (65)	0.60
Systolic blood pressure (mmHg)		125 ± 10	149 ± 13	<0.001
Diastolic blood pressure (mmHg)		70 ± 8	80 ± 9	<0.001
eGFR <60, *n* (%)		53 (22)	51 (24)	0.60
Albuminuria, *n* (%)		40 (17)	95 (46)	<0.001
*Pharmacological management*				
RAASi, *n* (%)		163 (68)	134 (64)	0.33
β-blockers, *n* (%)		115 (48)	93 (44)	0.42
Thiazide diuretics, *n* (%)		71 (30)	66 (31)	0.69
Calcium antagonists, *n* (%)		50 (21)	52 (25)	0.33
Loop diuretics, *n* (%)		52 (22)	29 (14)	0.03
Potassium sparing diuretics, *n* (%)		22 (9)	21 (10)	0.78
Number of antihypertensives		2 (1–3)	2 (1–3)	0.51
	No antihypertensive therapy, *n* (%)	39 (16)	44 (21)	0.85
	1 drug, *n* (%)	47 (20)	42 (20)	
	2 drugs, *n* (%)	64 (27)	45 (21)	
	3 drugs, *n* (%)	50 (21)	43 (21)	
	4 drugs, *n* (%)	29 (12)	27 (13)	
	5+ drugs, *n* (%)	10 (4)	9 (4)	
Hypertension requiring 4+ drugs, *n* (%)		39 (16)	79 (38)	<0.001
Total number of drugs		7.0 ± 2.6	6.7 ± 2.8	0.30
*Non-pharmacological management*				
BMI (kg/m^2^)		32.8 ± 5.8	32.9 ± 6.7	0.89
Serum 25 (OH) Vitamin D (nmol/L)		43 ± 20	41 ± 20	0.22
*Urinary excretion*				
Urinary creatinine excretion (mmol/day)		13.6 ± 4.9	14.1 ± 4.7	0.22
Urinary magnesium excretion (mmol/day)		3.9 ± 2.1	4.0 ± 1.9	0.43
Urinary phosphate excretion (mmol/day)		26.9 ± 12.3	28.2 ± 10.7	0.26
Sodium-to-potassium ratio (mmol/mmol)		2.5 ± 1.0	2.5 ± 0.9	0.49
*Calculated intake*				
Dietary salt intake (g/day)		10.7 ± 4.8	11.1 ± 4.4	0.47
Dietary potassium intake (g/day)		3.8 ± 1.3	4.0 ± 1.2	0.15
Dietary protein intake (g/day)		90 ± 29	93 ± 26	0.29

## Supplementary Materials

The following are available online at www.mdpi.com/2072-6643/9/7/709/s1, Table S1: Data collection in DIALECT.
