# Supplementary material for: Integrated Assessment of Pharmacological and Nutritional Cardiovascular Risk Management: Blood Pressure Control in the DIAbetes and LifEstyle Cohort Twente (DIALECT)
Source: Nutrients. 2017 Jul 6;9(7):709. doi: 10.3390/nu9070709 (PMC5537824; doi:10.3390/nu9070709)
Supplement: Supplementary file 1 [file nutrients-09-00709-s001.pdf]

Supplementary Table 1 – Data collection in DIALECT

Medical history

Medical history interview, review of electronic patient file

Medication use

Medication interview, drug overview from the pharmacist

Questionnaires

General

Smoking history, second hand smoking, occupation, nationality

Physical activity

Short QUestionnaire to ASses Health enhancing physical activity (SQUASH)

Dietary habits

Food-Frequency Questionnaire (FFQ)

Blood pressure

In a separate room, blood pressure is measured each minute during 15 minutes, while the patient is sitting in a supine position.

Physical examination

General

Height, weight, waist circumference, hip circumference

Neuropathy

Monofilament and VibraTip™

Body impedance

Bodyscan® Quadscan 4000 and the TANITA® BC418MA

Laboratory assessments

Serum

Hemoglobin, hematocrit, erythrocyte sedimentation rate, leukocyte and platelet count, c-reactive protein, total cholesterol, LDL- and HDL cholesterol, triglycerides, HbA1c, glucose, total bilirubin, aspartate aminotransferase, alanine aminotransferase, alkaline phosphatase, gamma-glutamyl transferase, albumin, total protein, N-terminal prohormone of brain natriuretic peptide, creatinin, eGFR (CKD-epi), urea, uric acid, sodium, potassium, calcium, phosphate, vitamin D, parathyroid hormone, magnesium, thyroid-stimulating hormone, free thyroxine, venous blood gas analysis, lactate

24h urine

Volume, creatinine excretion, sodium excretion, potassium excretion, calcium excretion, phosphate excretion, urea excretion, uric acid excretion, magnesium excretion, total protein excretion

Morning void urine

Dipstick test for erythrocytes, leucocytes, glucose, ketones, nitrite and pH

Albumine and albumine-to-creatinine ratio

Biobanking

Serum

10x serum, 9x EDTA, 1x EDTA + glutathion, 6x citrated, 10x heparin, 1x whole blood

24h urine collection

10x regular, 5x acidified to pH<2, 5x alcalized to pH>8

Single morning void collection

5x regular

Follow up

Continuous data

Blood pressure, Weight, Pharmacological treatment, HbA1c, LDL-cholesterol, eGFR, Urinary albumin-to-creatinine ratio

Endpoints

Macrovascular events

Microvascular events
Renal events
Mortality, all-cause and cardiovascular
